# Sonication-supported synthesis of cobalt oxide assembled on an N-MWCNT composite for electrochemical supercapacitors via three-electrode configuration

**DOI:** 10.1038/s41598-022-05964-8

**Published:** 2022-02-07

**Authors:** Rajendra Kumar Nare, Sivalingam Ramesh, Praveen Kumar Basavi, Vijay Kakani, Chinna Bathula, Hemraj M. Yadav, Prakash Babu Dhanapal, Rama Krishna Reddy Kotanka, Visweswara Rao Pasupuleti

**Affiliations:** 1 School of Applied Sciences, REVA University, Bangalore, 560064 India; 2 Department of Mechanical, Robotics and Energy Engineering, Dongguk University –Seoul, Pil-dong, Jung-gu, 04620 Seoul, Republic of Korea; 3 Department of Chemistry, Sri Venkateswara University, Tirupati, 517502 Andhra Pradesh India; 4 Department of Integrated System Engineering, School for Global Convergence Studies, Inha University, 100 Inha-ro, Nam-gu, 22212 Incheon, Republic of Korea; 5 Division of Electronics and Electrical Engineering, Dongguk University-Seoul, Pildong-ro 1 gil, Jung-gu, Seoul, 04620 Republic of Korea; 6 School of Nanoscience and Technology, Shivaji University, Kolhapur, 416004 India; 7 Department of Biomedical Sciences and Therapeutics, Faculty of Medicine and Health Sciences, University of Malaysia Sabah, Kota Kinabalu, 88400 Sabah, Malaysia; 8 Centre for International Collaboration and Research, Reva University, Rukmini Knowledge Park, Kattigenahalli, Yelahanka, Bangalore, 560064 Karnataka India

**Keywords:** Chemistry, Engineering, Materials science, Nanoscience and technology

## Abstract

The Co_3_O_4_@N-MWCNT composite was synthesized by a sonication-supported thermal reduction process for supercapacitor applications. The structural and morphological properties of the materials were characterized via Raman, XRD, XPS, SEM–EDX, and FE-TEM analysis. The composite electrode was constructed into a three-electrode configuration and examined by using CV, GCD and EIS analysis. The demonstrated electrochemical value of ~ 225 F/g at 0.5 A/g by the electrode made it appropriate for potential use in supercapacitor applications.

## Introduction

Electrochemical capacitors are supercapacitors that can afford power densities superior to those of other electrochemical capacitor systems. Supercapacitors maintain great advantages in fabricating electrodes involving GCD cycles and excellent cyclic stability^1–3^. Supercapacitors are divided into two types, electric double-layer capacitors (EDLCs) and pseudocapacitors, based on their energy storage mechanism. In the EDLC process, the energy is collected by the electrostatic adsorption of charges on the electrode surface related to the parallel plate capacitor. On the other hand, the energy in the pseudocapacitor is collected by the reversible Faradaic redox reaction involved in the electrode surface^4–6^. Carbon nanomaterials have been widely employed in fabricating supercapacitor electrodes owing to their significantly improved chemical, mechanical electrical, and electrochemical behaviours, including their noteworthy surface properties. In particular, carbon nanotubes are receiving collective scientific interest due to their notable thermal stability in addition to the above-described advantages. During the last decade, extensive research has focused on carbon nanotubes to explore their application as sensors, supercapacitors, and electrocatalysts due to their high chemical stability, large volume ratio, and excellent electrochemical benefits^7,8^. The synergetic effects (or) strong interaction of carbon nanotubes functional groups with cobalt oxides have shown promising results in the fields of gas sensors, supercapacitors, and electrocatalysts^9–13^. To achieve pseudocapacitance, either transition metal oxides or conducting polymers or both together are used to fabricate the supercapacitor electrodes. Of the metal oxides, cobalt oxide (Co_3_O_4_) has gained an appropriate position compared to other electrode systems. The enhanced surface area, facile electrode fabrication, better chemical stability, and outstanding morphological properties associated with cobal oxide-based materials find their application in the field of supercapacitors^13–15^. In addition, Co_3_O_4_ materials are also used as working electrodes for sensors, heterogeneous catalysts, electrochromic devices, and magnetic materials^16^. It has been reported that morphological patterns such as mesopores, nanocubes, nanospheres and nanorods exert different impacts on the fabrication of electrode materials^17^. Apart from morphological influence, crystal size, aspect ratio, orientation, and crystalline density also play a vital role in the enhancement of electrochemical phenomena^17^.

Aligned Co_3_O_4_ nanowires on nickel foam electrode materials synthesized by a hydrothermal process achieved a capacitance of ~ 750 F/g at 0.5 A/g. The flower-like nanostructure of cobalt oxides/carbon electrode materials registered a capacitance of ~ 330 F/g at 0.5 A/g in the solvothermal process^18^. The porous morphology of the Co_3_O_4_ film synthesized via electrode deposition showed a maximum capacitance of ~ 443 F/g at 0.5 The nanocrystalline morphology of Co_3_O_4_ material-based electrodes exhibited improved electrochemical properties with improved cyclic stability^19–22^.

The carbon nanotubes (CNTs) are widely used as support for active metal nanoparticles for catalytic properties due to thier outstanding resistance to challenging the reaction and surface properties for chemical functalization and nitrogen doping process. These surface functional groups can be used to modify the catalytic performance of the metal nanoparticles for various potential applications. In particular, the N-MWCNTs are found to be a auspicious support for catalysis and improved electrochemical properties^23–25^. A higher dispersion of the supported metals can be accomplished on N-MWCNTs than on nitrogen-free CNTs, which was attributed to a higher amount of surface nucleation sites and to the formation of some individual sections around the N-rich sites, allowing efficient anchoring of metal nanoparticles for various potential applications.Frackowiak et al. reported that the specific capacitance of MWCNTs was increased from (80 to 135) F g^[−1 26^, while treating MWCNTs with acid electrolyte and other KOH electrolytes is almost 90 F g^−1^. The pristine cobalt oxide is not constant value of the specific capacitance, the outcome values changes accordingly the morphology and electrolyte. The specific capacitances almost is (~ 150–225) F g^−1^ in the nanocrystalline morphology with KOH electrolyte^27–29^. The following publications showed different outcome values of cobalt oxides with different approach of the electrochemical reaction. The following reports were increased or decreased the specific capacitances, cyclic stability depends on the synthetic route, morphologies of the materials and types of electrolyte. Based on the literature, we synthesised Co_3_O_4_@N-MWCNT composite via sonication assisted thermal reduction process and the outcome specific capacitance 225 F/g at 0.5 A/g with excellent retention in the 5000 cycles. Therefore, the synthesized composite materials useful for supercapacitor application with excellent cyclic retention. These surface functional groups can be used to tailor the catalytic performance of supported metal nanoparticles for various potential applications. Nitrogen-doped carbon nanotubes (N-MWCNT) are found to be a promising support for hydrogenation catalysts and amended the electrochemical properties. A higher dispersion of the supported metals can be accomplished on N-MWCNTs than on nitrogen-free CNTs, which was attributed to a higher amount of surface nucleation sites and to the formation of some individual sections around the N-rich sites, allowing efficient anchoring of metal nanoparticles for various potential applications^30–33^.

Based on the amended benefits accompanied by Co_3_O_4_-based materials, we designed and synthesized cobalt oxide@nitrogen-doped multiwalled carbon nanotube composite by sonication-mediated thermal reduction processes and explored their supercapacitor applications. This composite displayed a high specific capacitance of ~ 225 F/g at 0.5 A/g and excellent cyclic stability in the presence of a 3 M KOH electrolyte.

## Experimental methods

### Materials

Multiwalled carbon nanotubes, cobalt acetate tetrahydrate [Co(CH_3_COO)_2_·4H_2_O], potassium permanganate (KMnO_4_), hydrochloric acid (HCl), sulfuric acid (H_2_SO_4_), phosphoric acid (H_3_PO_4_), ammonia (NH_3_, 30%), absolute ethanol (C_2_H_5_OH) and hydrogen peroxide (H_2_O_2_), were acquired from Sigma-Aldrich chemicals, and all electrochemical studies were carried out by employing double distilled water.

### Co_3_O_4_@N-MWCNT synthesis

**Figure 1:**
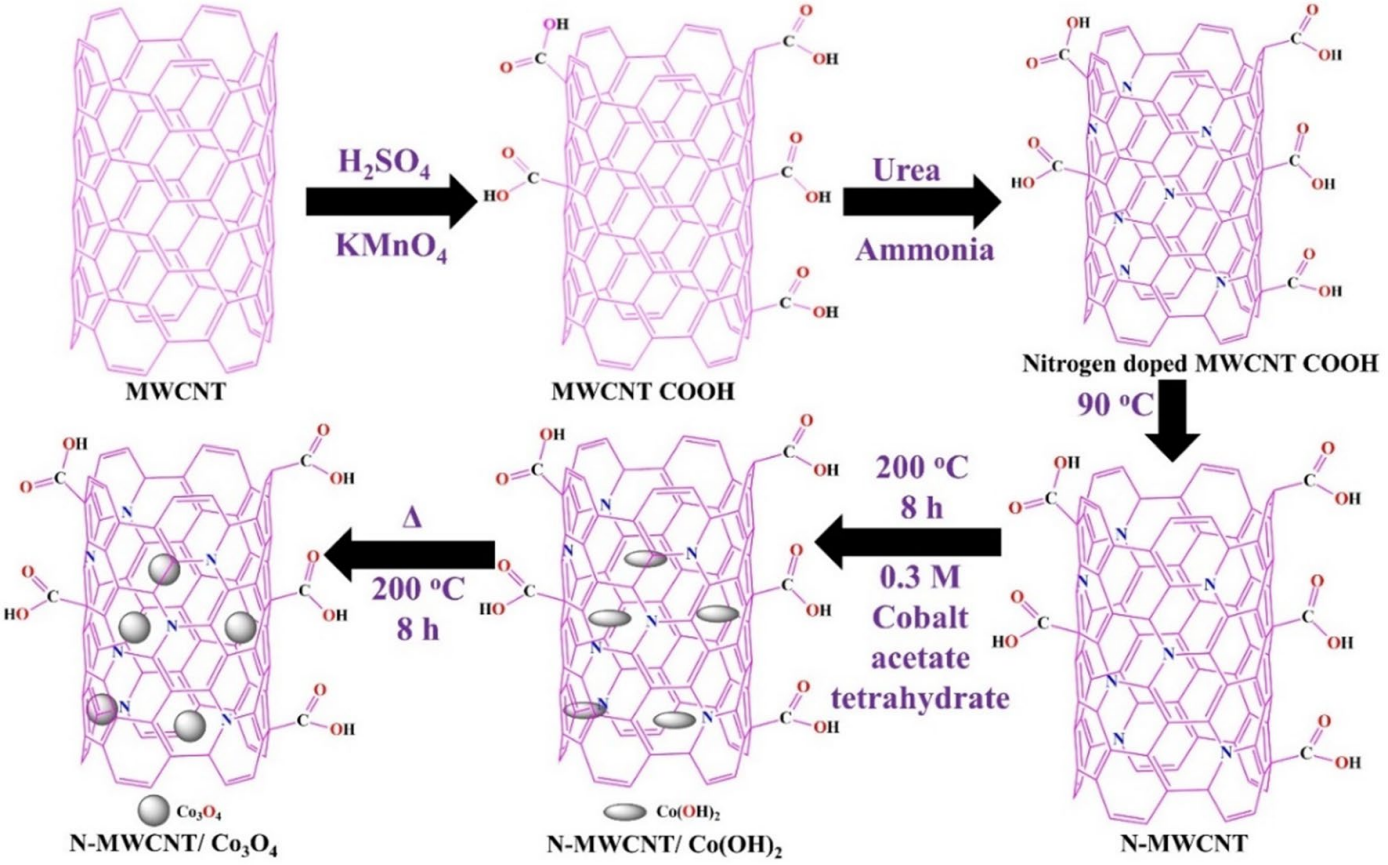
Schematic representation of Co_3_O_4_@N-MWCNT composite synthesis by thermal reduction process.

In a typical synthesis of a Co_3_O_4_@N-MWCNT composite, 0.8 g of nitrogen-doped MWCNTs was diffused in 200 ml of double DD water via sonication for 2 h. To this end, 0.3 mol of cobalt acetate tetrahydrate and Co(CH_3_COO)_2_·4H_2_O were added, followed by 20 mL of 30% ammonia, and the whole solution was roused at 90 °C for 12 h. At this point, the whole reaction combination was transferred to an autoclave reactor, and the thermal reduction process was carried out at 200 °C for 8 h. The preceipitated Co_3_O_4_@N-MWCNT composite material was filtered and washed with a 1:1 solution of DD water/ethanol repetitively and purified at 95 °C for 12 h. This dried composite was stored in an airtight bottle and subjected to structural, morphological, and electrochemical studies. Schematic illustration of synthetic protol for Co_3_O_4_@N-MWCNT composite is shown in Fig. 1.

### Fabrication of electrodes for supercapacitor study

The composite was fabricated via a three-electrode configuration, and electrochemical studies were performed by CV, GCD, and EIS analysis. The composition of active material (80 wt%), 10 wt% conducting carbon black as the conductive agent and 10 wt% polyvinylidene fluoride (PVDF) binder were mixed with N-methyl pyrrolidinone (NMP) via sonication to complete the uniform slurry. After that, this composite slurry was coated uniformly on a strip of nickel wire (1 × 1 cm^−2^) current collector and dried in an oven at 90 °C for 10 h. Then, the slurry was coated on the nickel wire for the working electrode in the CV analysis. counter electrode is (platinum wire) and reference electrode (Ag/AgCl) for electrochemical studies.

### Materials characterization

The synthesized Co_3_O_4_@N-MWCNT composite material was analysed by using a He–Ne laser beam in RM 200 Raman spectral microscopy. XRD results were characterized via a Rigaku Rotaflex (RU-200B) X-ray diffractometer. The morphological properties and SAED pattern of the composite samples were studied by FE-SEM (Hitachi S-48000), FE-TEM; JEOLJEM-2010F. Thermo Scientific, USA) combined with an Al Kα source (100 to 3 keV) by X-ray photoelectron spectroscopy examination of the composite elemental composition. CHI 7081C (CH Instruments, workstation Inc., USA) electrochemical characterization via a three-electrode configuration for supercapacitor study.

## Results and discussion

**Figure 2:**
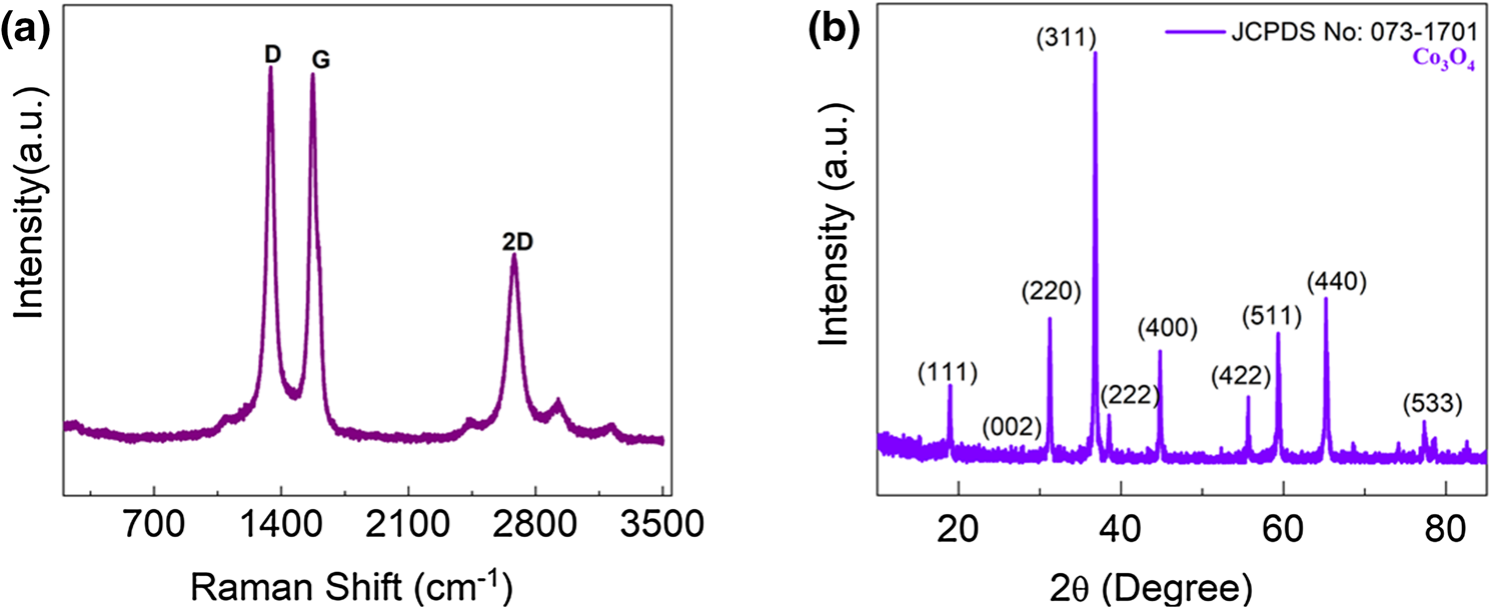
Composite confirmation of (**a**) Raman and (**b**) XRD results.

The Raman spectra of the Co_3_O_4_@N-MWCNT composite results are shown in Fig. 2a. The Raman shifts at ∼1340, ∼1572, and 2675 cm^−1^ can be attributed to the three distinct types of peaks of the N-MWCNT composite. The D band is attributed to the lattice defect that highlights the phonon mode of vibration from the N-MWCNT surface. The G band represents the C–C (vibrational modes), and E_2g_ symmetry denotes the doubly degenerated phonon modes^23^. Generally, for the ID/IG values of N-MWCNTs, there was a small increase in intensity from 0.95 to 1.35, which proposes partially ordered crystal structures modified on the carbon surface. Furthermore, the composite I_D_/I_G_ values decreased by ~ 0.95 due to the decreasing sp^2^ domain size of the carbon surface from the N-MWCNT materials. The Raman technique was effectively used to explore the Co_3_O_4_ nanostructure (450–673) cm^−1^ obtained from its cobalt acetate precursor. Therefore, the intensity and interaction of Co^2+^ and the N-MWCNT composite depend on the concentration of cobalt acetate in the aqueous solution by the thermal reduction process.

The XRD results explain the crystallinity, structural properties, and phase purity of the composite material, and the diffraction results are shown in Fig. 2b. From the XRD results, the diffraction peaks at 2θ = 18.78°, 31.24°, 36.93°, 38.47°, 44.76°, 55.81°, 59.47°, 65.28°, 68.50°, 74.18°, 77.16°, and 78.46° were smoothly marked as (111), (220), (311), (222), (400), (422), (511), (440), (531), (620), (533), and (622) of the cubic spinel structure of Co_3_O_4_ and matched with JCPDS No.:65-3103. The nanoparticle size of the cobalt oxides asertained by FE-TEM results was consistent with XRD analysis. The crstallinty sizes of the cobalt oxide nanoparticles increase with increasing calcination temperature, controlling composite formation for supercapacitor applications^24^.

**Figure 3:**
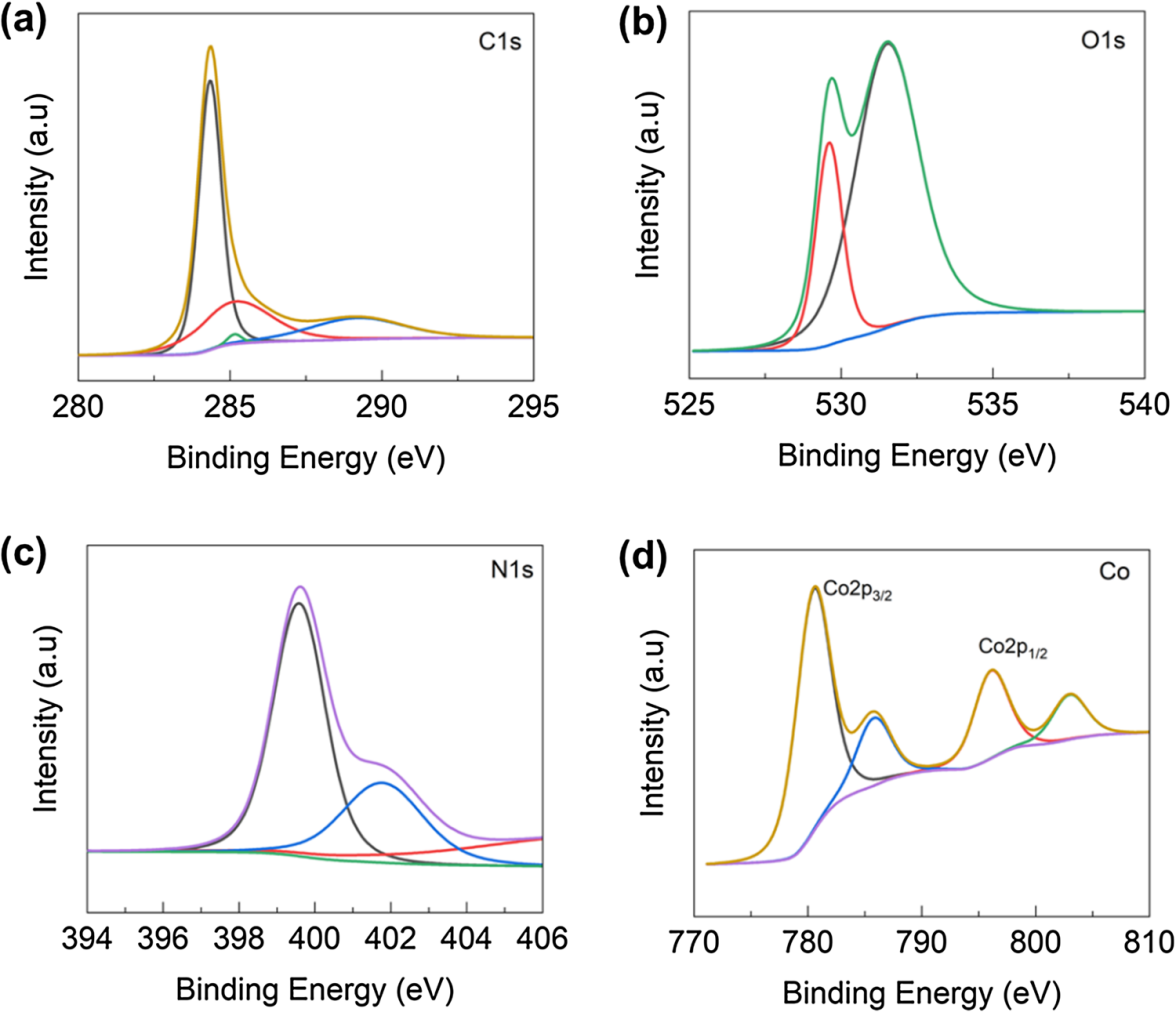
XPS studies of (**a**) C1s, (**b**) O1s, (**c**) N1 s and (**d**) Co2p, of Co_3_O_4_@N-MWCNT composite materials.

XPS results show the elemental composition, electronic structure, and oxidation state of the composite materials. Figure 3a–d demonstrates the core level spectrum of the Co 2p state of the Co_3_O_4_ composite at 200 °C via the thermal reduction process. The two main peaks of cobalt oxides at 780.48 and 796 eV correspond to the octahedral Co^3+^ and Co^2+^ (tetrahedral) structures, respectively. The low-intensity peak at ~ 796 eV is attributed to the characteristic Co_3_O_4_ phase^25^. The peaks centered at 529.61 and 531.52 eV can be assigned to the signs of the four oxygen types, indicating the effective synthesis of Co_3_O_4_ nanostructures in the composite. The other peaks at 399.9 (N 1s) and 284,288 (C 1s) peaks are confirmed in the composite.

**Figure 4:**
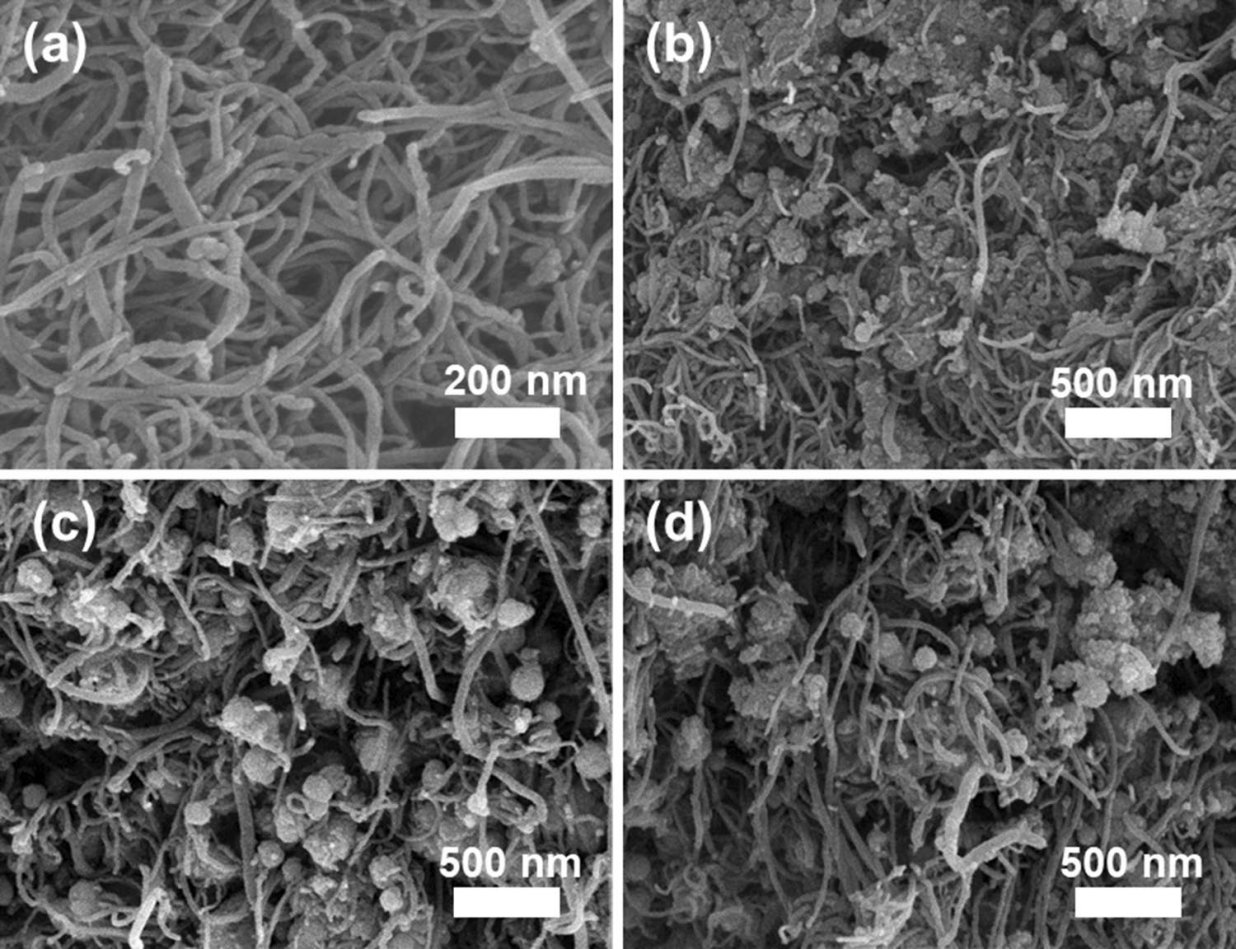
SEM analysis of (**a**) N-MWCNT and (**b**–**d**) N-MWCNT/cobalt oxide composite.

**Figure 5:**
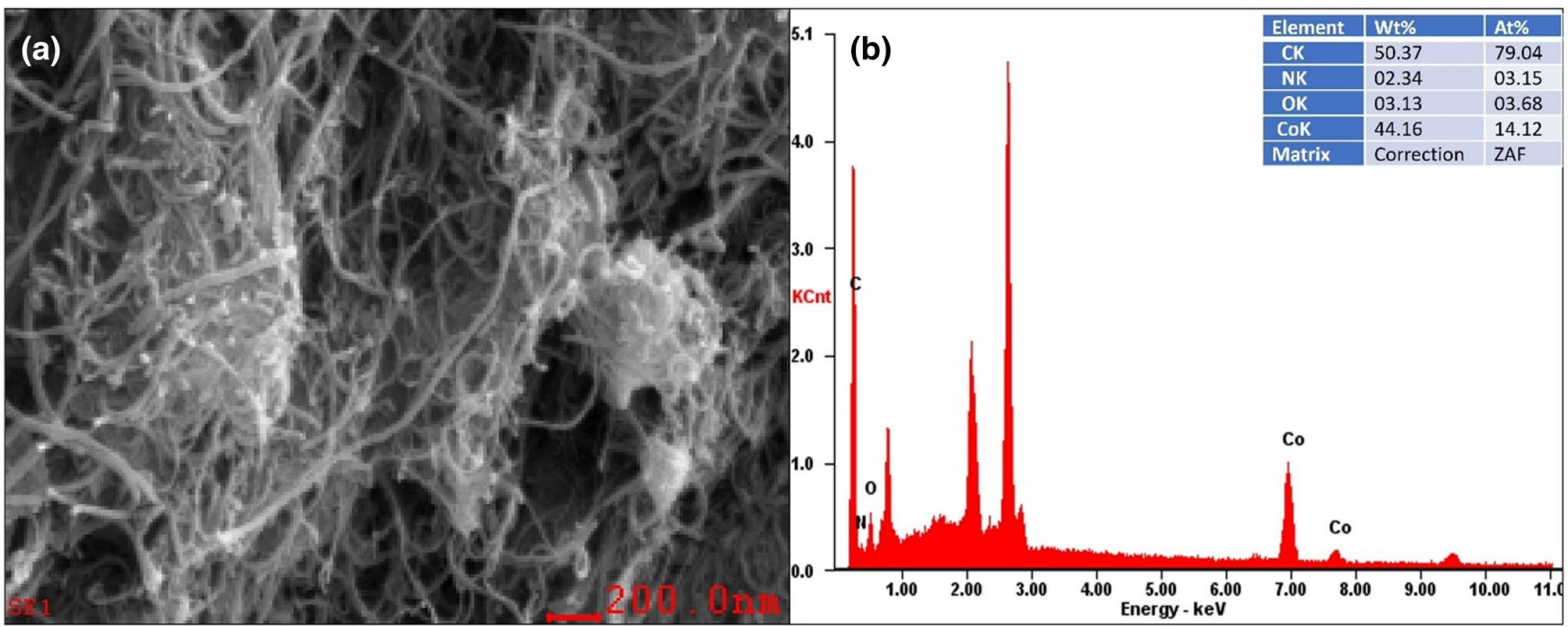
SEM–EDS analysis of the (**a**) N-MWCNT and (**b**) SEM–EDS peaks of N-MWCNT/cobalt oxide composites.

**Figure 6:**
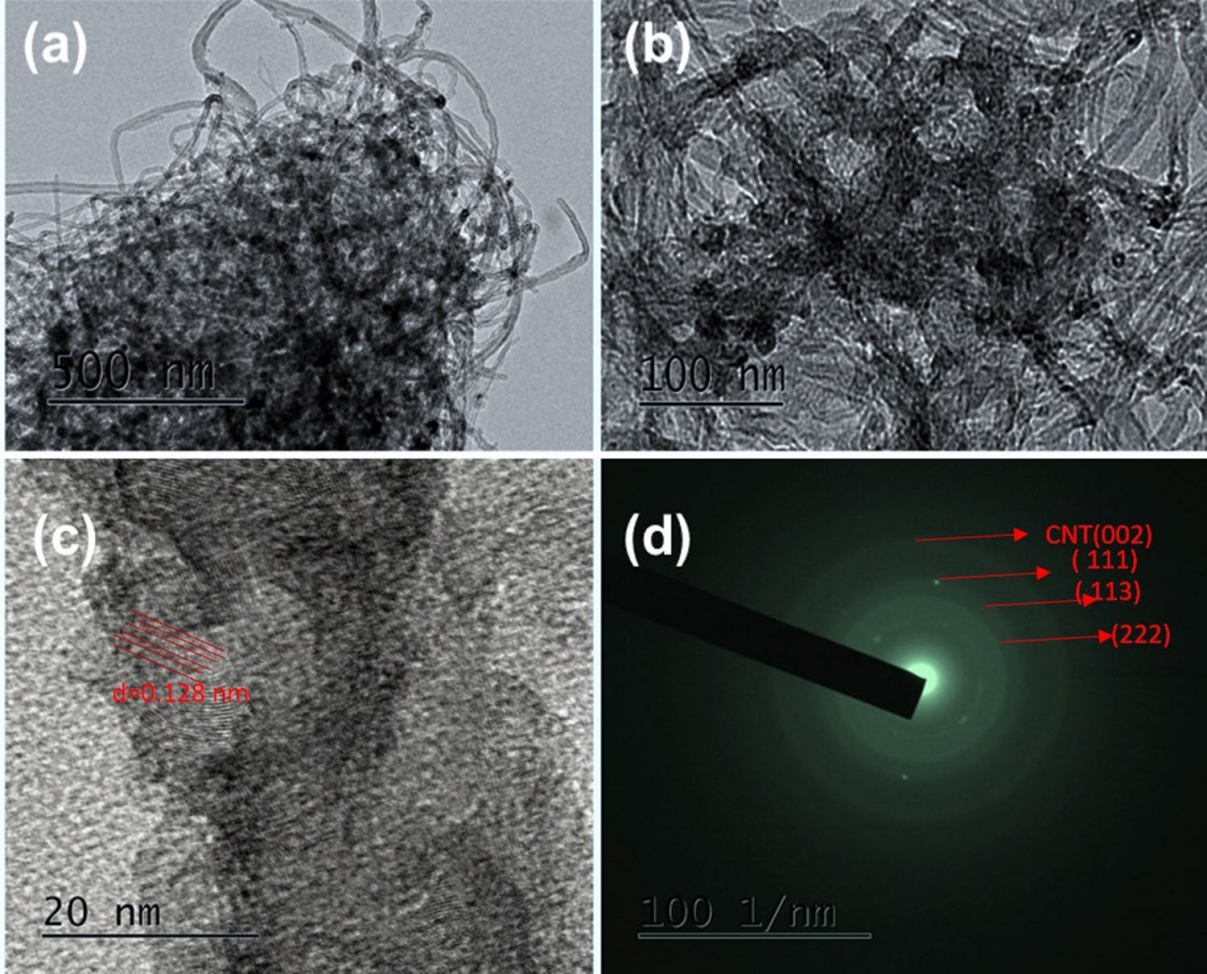
(**a**–**c**). HR-TEM morphology and (**d**) SAED image of N-MWCNT materials.

**Figure 7:**
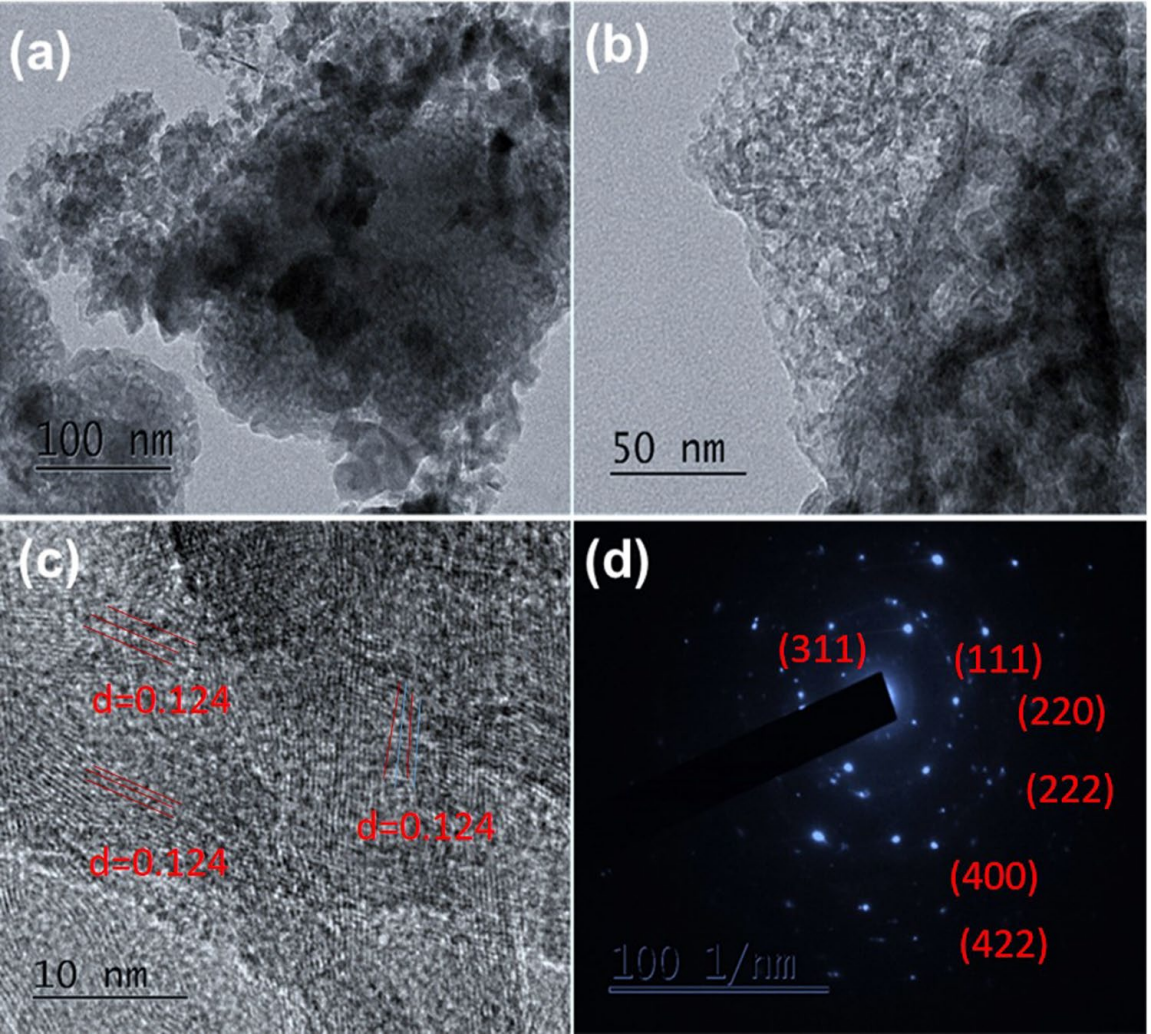
HR-TEM morphology of (**a**–**c**) images and (**d**) SAED pattern of Co_3_O_4_@N-MWCNT composite materials.

The surface morphological properties of the composite were studied by SEM, SEM–EDS and HR-TEM, and the acquired results are shown in Figs. 4, 5, 6, and 7. This reveals that the N-MWCNT tubes exhibit a well-ordered arrangement with an outer diameter of ~ 30–40 nm and an inner thickness of ~ 10–20 nm. Figures 6d and 7d represent the SAED pattern of nanotubes and composite materials to confirm the nanocrystalline structured composite. The SEM–EDS morphology of the elements confirmed that the C, O, N and cobalt oxides in the composite materials are shown in Fig. 5a,b.

**Figure 8:**
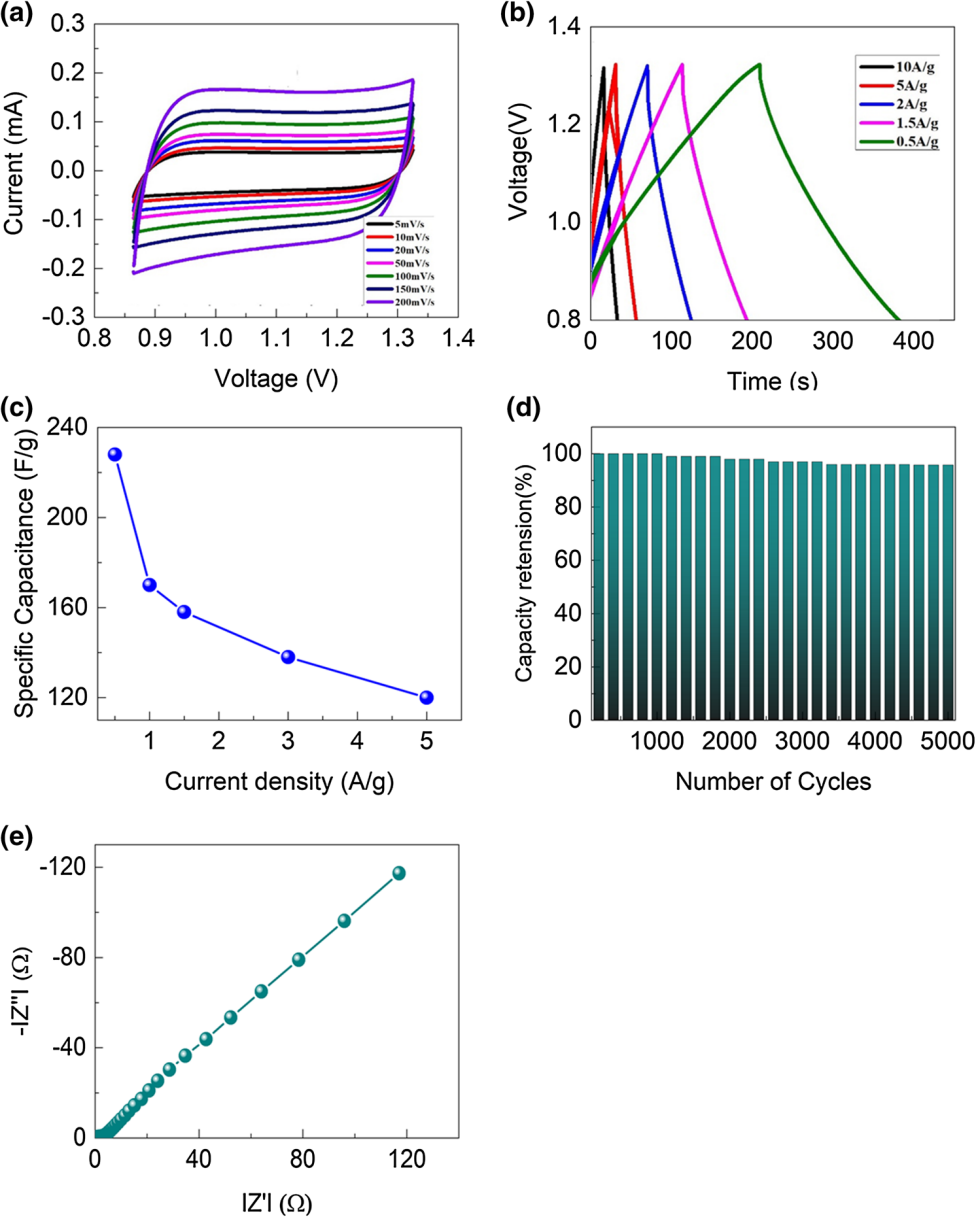
(**a**) CV, (**b**) GCD, (**c**) variation in current density (Vs) specific capacitance, (**d**) number of cycles and (**e**) EIS results of the composite materials.

The electrochemical properties of various nanostructured cobalt oxides and N-MWCNT material electrodes were studied for supercapacitor applications^26,27^. Furthermore, the electrical properties of the composite were investigated via three-eleectrode configuration by using CV, GCD, and EIS analysis. In this configuration, the working electrode (Co_3_O_4_@N-MWCNT, reference electrode (Ag/AgCl) and Pt act as counter electrodes in the presence of 3 M KOH electrolyte at room temperature. The CV results of the synthesized composite electrodes at different applied scan rates are shown in Fig. 8a–e. Electrochemical CV curves (Fig. 8a) are obtained for 5, 10, 20, 50, 100, 150, and 200 mV/s with a potential window from 0.8 to 1.4 at different applied scan rates. In this result, well-defined rectangular peaks were observed due to the enhancement of the electrochemical properties of the composite materials. While increasing the scan rates, the peak intensities increase towards higher potentials. This is corroborated by the rapid electrochemical charge–discharge obtained between the active composite material and electrolyte surface, as shown in reactions 1 and 2. Generally, the rate competence was mainly reliant on three routes: (i) electrolyte and ion diffusion, (ii) electrode surface, and ion adsorption, and (iii) charge transfer and electrode. Based on the three steps, with increasing scan rate, the reaction rate relatively lowers and decreases the specific capacitance. Therefore, the characteristic CV curves did not change significantly, which indicates that the Co_3_O_4_@N-MWCNT composites have a unique rate capability of the electrode materials and its electron transfer reaction in detail^27^.

Cobalt oxides are involved in charge transfer or electron transfer reactions in the presence of a 3 M KOH electrolyte. The resulting anodic and cathodic curves were defined as the electrochemical EDLC behaviour of the materials in the CV analysis. The reaction mechanisms are connected with previously reported cobalt oxide materials via different electrolytes. The CV peaks significantly vary on the mode of morphological, surface, and structural properties of composite materials. Therefore, cobalt oxides are potential materials involved in electrochemical reactions for pseudocapacitance applications^28,29^. Figure 8b demonstrates the GCD data of the composite results and indicates symmetrical triangular curves at current densities of 0.5, 1, 1.5, 2,5 and 10 A g^−1^, which signifies EDLC behaviour in the fixed potential range of (0.8 to 1.4) V. Increasing the applied current density above 10 A/g reduces the EDLC behaviour and increases the resistance properties. Hence, the GCD curves after 10 A/g diverging linearity indicate a decrease in the EDLC behaviour of the composite. Therefore, the nature of the GCD profile of the composite indicates the diffusion of ions or migration of the ions via a 3 M KOH electrolyte and increases the electrochemical capacitance. The electrochemical capacitance (~ 225 F g^−1^) of the pristine N-NMWCNT and Co_3_O_4_ electrode materials. This increase in the specific capacitance values depends on the electronic conductivity, redoxy activity and size of the nanoparticles in the composite^30–33^. The capacitance was 225 F g^−^1 at a current density of 0.5 A g^−1^, demonstrating the unique rate capability via the electrolyte. Figure 8c proves the plot of specific capacitance (Vs) for different current densities of the electrode materials. The resulting capacitances are ~ (225,168, 156, 135, and 122) F g^−1^, and different current densities are applied from (0.5 to 10) A g^−1^. The outcome specific capacitance is depending on the morphology, surface area of the materials and electrolyte variation. In the cobalt based carbon systems showed nearly (280 to 450) F/g at 0.5 A/g. Therefore, our reported composite materials showed excellent cyclic retention capabilities in presence of KOH electrolyte.The various synthetic approaches are increasing the specific capacitances and cyclic stability and retention.Rahul Kumar et al. (2021) was synthesized carbon coated cobalt oxide electrode for supercapacitor applications. The electrode materials showed the specific capacitance value is almost 395 F/g at 5 Mv/g in presence of 1 M KOH electrolyte.Madhu Gaire et al. (2021) reported on the preparation of cobalt oxide –RGO supercapacitor electrode by photo thermal processing. The fabrication of the electrode shows the specific capacitance is 69 F/g at 1.6 A/g with 30,000 cycles.Mojtaba Mirzaeian et al. (2020) reported on the Improvement of the Pseudo capacitive performance of cobalt based electrodes for electrochemical capacitors. The working electrode was prepared by pyrolysis of aerosol from the cobalt acetate. The electrode showed the specific capacitance of ~ 509.6 F g^−1^ at a current density of 0.38 A g^−1^.Niveditha et al.(2018) reported on the synthesis of Feather like highly active Co_3_O_4_ electrode for supercapacitor application: a potentiodynamic approach. The electrode materials showed the specific capacitance 367.67 F g^−1^ at scan rate of 20 mv sec^−1^ with 1600 cycles. Liu et al. (2017) reported on Facile synthesis of ultrafine cobalt oxide nanoparticles for high-performance supercapacitors. In this work, the outcome specific capacitance is almost 523 F g^−1^ at 0.5 A g^−1^ with 1500 cyles. The comaparision of electrochemical properties of the various syntheisezd composite electrode materials are shown in Table 1.

**Table 1:** Comparison of the electrochemical results of cobalt oxide-based composite electrodes reported in the literature.

Electrode material	Preparation method	Capacitance (F g^−1^)	Cyclic stability	Ref
Co_3_O_4_ symmetrical electrode	Magnetron sputtering	392 F g^−1^ at 2 mA cm^−2^	No loss after 10,000 cycles	^40^
Co_3_O_4_/MWCNT/graphene nanotube electrode	Ultrafast microwave irradiation	600 F g^−1^ at 0.7 A g^−1^	5.5% loss after 5000 cycles	^41^
Co_3_O_4_/graphite electrode	Co-precipitation method	239.5 F g^−1^ at 0.5 A g^−1^	2.68% loss after 1000 cycles	^42^
Co_3_O_4_/MWCNT carbon composite	Chemical deposition method	273 F g^−1^ at 0.5 A g^−1^	22% loss after 500 cycles	^43^
Co_3_O_4_/CNT nanocomposite	Molecular level mixing process by ultra-sonication	525 F g^−1^ at 0.5 A g^−1^	19.5% loss after 2000 cycles	^44^
Co_3_O_4_@N-MWCNT	Sonication supported hydrothermal synthesis	225 F g^−1^ at 0.5 A g^−1^	2.2% loss after 5000 cycles	Present study

The cyclic stability of the composite electrode (Fig. 8d) shows that 0.5 A/g for 5000 continuous GCD cycles and 2.2% loss occurred, representing excellent cyclic stability in the strong electrolyte 3 M KOH. Figure 8e demonstrates the EIS results of the composite materials. This result authorizes the electrochemical properties of the materials at low frequencies with semicircles in the high-frequency region. The consequent (Fig. 8e) lower resistance (Rs) and charge transfer (Rct) proved the electrochemical enhancement of the composite materials. The electrochemical properties of the composite materials show Rs and Rct ~ 0.7 and 35, respectively. This increase in the electrochemical properties of the composite electrode is useful for supercapacitor properties^30–33^.

The specific capacitance of the synthesized composites was comparable to considerably increases in the stability of cobalt oxide-based composite electrodes reported previously (Table 1)^40–44^. Although it is challenging to arrange a clear-cut comparison of the electrode materials, the data in Table 1 were recorded using a range of parameters, such as the fabrication of electrodes, synthesis methods, capacitances observed at different current densities/scan rates, and cyclic stabilities^45–50^. In the present work, the composite demonstrated excellent electrochemical performance compared to the electrodes reported previously due to the good capacitance at a higher current density of 0.5 A g^−1^ and the high cyclic stability, losing only 2.2% of its initial capacitance after 5000 cycles. The resulted morphological and electrochemical behaviour of the composite materials are compared to previously syntheized electrodes published in the literature^45–50^.

## Conclusion

The synthesized Co_3_O_4_@N-MWCNT composite materials were constructed in a three-electrode configuration for supercapacitor applications. The capacitance value and cycling stability were enhanced up to 5000 cycles with a retension of 97.8% (2.2% loss at 0.5 A g^−1^). The enhancement of the composite electrode was due to the electrolyte distribution and morphological, surface and controlled synthesis, which was developed for supercapacitor applications. The composite electrode exhibited a marked specific capacitance (~ 225 F/g at 0.5 A/g), exceptional cyclic stability, improved morphological properties, and excellent cyclic retention.
